# PCR-DGGE Analysis Proves the Suppression of *Rhizoctonia* and *Sclerotium* Root Rot Due to Successive Inoculations

**DOI:** 10.3390/jof8020133

**Published:** 2022-01-28

**Authors:** Mohsen Mohamed Elsharkawy, Shuhei Kuno, Mitsuro Hyakumachi, Yasser S. Mostafa, Saad A. Alamri, Sulaiman A. Alrumman

**Affiliations:** 1 Agricultural Botany Department, Faculty of Agriculture, Kafrelsheikh University, Kafrelsheikh 33516, Egypt; 2 Laboratory of Plant Pathology, Faculty of Applied Biological Sciences, Gifu University, 1-1 Yanagido, Gifu City 501-1193, Japan; virusb007@yahoo.com (S.K.); hyakumac@gmail.com (M.H.); 3 Department of Biology, College of Science, King Khalid University, Abha 9004, Saudi Arabia; ysolhasa1969@hotmail.com (Y.S.M.); amri555@yahoo.com (S.A.A.); salrumman@kku.edu.sa (S.A.A.)

**Keywords:** *Rhizoctonia solani*, *Sclerotium rolfsii*, binucleate *Rhizoctonia*, *Trichoderma* spp.

## Abstract

The soil-borne pathogens *Rhizoctonia solani* and *Sclerotium rolfsii* have emerged as major pathogens of radish (*Raphanus sativus*) worldwide. The induction of soil suppressive of radish root rot disease was evaluated in soil repeatedly inoculated with *R. solani*, nonpathogenic binucleate *Rhizoctonia* sp. AG-A W1 (BNR) and *S. rolfsii*. The repeated inoculations of soil with *R. solani* and BNR significantly suppressed the disease severity of *R. solani* and *S. rolfsii* compared to the control. In contrast, the repeated inoculation of soil with *S. rolfsii* significantly suppressed only the pathogen, *S. rolfsii*. The community structure was examined using PCR-DGGE (polymerase chain reaction denaturing gradient gel electrophoresis) method. The bands of *Trichoderma* sp. were observed in the first, second and third inoculations of the soil with BNR. Similarly, bands of *Trichoderma* sp. were observed in the second and third inoculations of the soil with *S. rolfsii* and *R. solani*. Compared to the control, disease severity was significantly reduced in the soil repeatedly inoculated with *S. rolfsii* and *R. solani* . In conclusion, *Trichoderma* species were accumulated in specific patterns depending on the applied fungal inoculum in the suppressive soil.

## 1. Introduction

Crops are seriously affected by soil-borne plant pathogens. Root rot is a common disease found in the soil of high-humidity locations. *Rhizoctonia solani* and *Sclerotium rolfsii* are the most dangerous pathogens for radish root rot disease. *R. solani*, a phytopathogenic fungus, is categorized into anastomosis groups (AGs) based on their hyphal anastomosis reactions [1]. Brown blotches, damping off in seedlings and root rot are a few of the economically important plant diseases caused by the fungus. *R. solani* strains vary in their host specificity and virulence levels toward their plant hosts. On the other hand, the pathogen *S. rolfsii* infects around 500 plant species and causes significant losses in global crop production. *S. rolfsii* is found all over the globe; however, it is most common in tropical and subtropical environments [2].

In general, continuous cropping of the same crop increases soil-borne diseases due to increased concentrations of pathogens in the soil. However, this phenomenon occurs gradually. The disease decline phenomenon was reported in several diseases, such as the “take-all-decline (TAD)” caused by *Gaeumanomyces graminis* var. *tritici* [3], potato scab disease caused by *Streptomyces scabies* [4] and sugar beet root rot caused by *R. solani* AG2-2 [5,6]. Disease decline soil is a type of soil that suppresses disease. Disease does not occur even if the soil is inoculated with a new inoculum of the pathogen [7]; therefore, this mechanism could be used as a biological control. Similarly, suppressive soil was also artificially induced by repeated pathogen inoculations in the field. Wildermuth [8] reported that soil inoculation with *G. graminis* var. *tritici* (Ggt) suppressed not only the disease caused by Ggt, but also other wheat diseases caused by *Phialophora*-like pathogenic fungi Ggt. The author also reported a new biocontrol agent for Ggt, *G. graminis* var. *graminis* (Ggg), which is now common for biological control of *Phialophora*-like pathogens and filamentous fungi of Ggt. Disease decline of *R. solani* was induced in the soil by the continuous cropping of radish in soil inoculated with *R. solani* AG-4 under greenhouse conditions [9,10,11,12,13,14,15]. In addition, suppression of root rot disease in wheat, caused by *R. solani* AG-8, was reported after inoculation of soil with *R. solani* AG-8. Meanwhile, no inhibition for *R. solani* AG-8 was observed in soil inoculated with *R. oryzae* [16]. The prevalence of antagonistic microorganisms was reported to depend in many cases on the suppressive effect of the soil. These microorganisms parasitize the hyphae [12], kill sclerotia and suppress hyphal growth [6]. In general, *Trichoderma* spp. were reported to be significantly involved in the development of suppressive soil [12,13]. However, in suppressive soils, the patterns of antagonistic microbial accumulation have not yet been well elucidated.

Here, the suppressive soils were generated by repeated inoculation with BNR, *R. solani* and *S. rolfsii*. The population dynamics of soil microorganisms were examined using a polymerase chain reaction-denaturing gradient gel electrophoresis (PCR-DGGE) method. PCR-DGGE is one of the most common fingerprinting techniques. It is an efficient, non-time-consuming strategy for processing large sets of clones [17]. Schabereiter-Gurtner et al. [18] suggested a method to identify the representative bands observed by DGGE. The primer pair 27F and 1497R was used to amplify the 16S rDNA sequences [19]. Additionally, DGGE was used to identify bacterial flora in different food products [20,21,22]. Recently, attempts have been made to analyze the soil microbial community by using PCR-DGGE [23]. DNA (extracted from soil microorganisms) was amplified by PCR and electrophoresed on polyacrylamide gel. This method for separating DNA fragments can measure the microbial community structure in the sample, including microorganisms that are difficult to culture [24]. Therefore, this study aims to determine the population dynamics of some soil microorganisms in radish. *Trichoderma* spp. as antagonistic fungi were identified. Theirexistence , distribution and role were assessed. The accumulation patterns of soil microorganisms by PCR-DGGE in repeatedly inoculated soil with *S. rolfsii*, *R. solani* and BNR were confirmed.

## 2. Materials and Methods

### 2.1. Preparation of Fungal Inocula

The plant pathology department of Gifu University provided the isolates; non-pathogenic binucleate *Rhizoctonia* AG-A W1, *Rhizoctonia solani* AG1-IC RH28 and *Sclerotium rolfsii* SR0205 [25,26,27]. Isolates were mass cultured on autoclaved barley grain as described by Elsharkawy et al. [15,28]. Both of *S. rolfsii* and *R. solani* were highly pathogenic to radish plants. Additionally, sterilized barley grains using the same procedures served as control. All isolates were maintained on potato dextrose agar (PDA, Becton, Dickinson and Company, Sparks, NV, USA) at 4 °C with a periodic transfer.

### 2.2. Soil Inoculations and Pathogenicity Test

Soil was repeatedly inoculated with BNR, *S. rolfsii* and *R. solani* , and the pathogenicity of radish root rot was investigated. A mixture of sandy loam soil from the Gifu University research field and Yahagi sand 1:1 (*w*/*w*) was prepared and filtered through a sieve (mesh 5 mm). The soil was mixed with inocula of BNR, *R. solani* and *S. rolfsii* at 0.5% (*w*/*w*) and packed in plastic pots (6 cm × 8 cm). Sterilized barley grains were mixed with the soil in the control treatment. Ten seeds of radish (*Raphanus sativus* L.) were grown in each pot. Pots were incubated in the greenhouse at 23–27 °C and 12 h photoperiod using fluorescent bulbs at 250–300 µE m^–2^ s^–1^. The seedlings were irrigated to keep moisture and were evaluated after 2 weeks. After removing the seedlings and debris, the soils were homogenized, and 10 g soil was sampled for microbial population analysis. The remaining soil was then re-inoculated with 0.5% (*w*/*w*) barley grain containing fungal inocula before sowing radish seeds. The soil was re-inoculated twice as described above (3 inoculation times in total). The experiment was repeated three times and disease severity was assessed. Disease severity was assessed using a disease severity index as follows: 0, no symptoms (healthy seedlings); 1, <50% of the hypocotyls covered with brown lesions; 2, >50% of the hypocotyls covered with dark brown lesions; 3, seedlings dead after germination; 4, no germination. The following formula was used to calculate the DS values:

DS value = [(0 × N_0_) + (1 × N_1_) + (2 × N_2_) + (3 × N_3_) + (4 × N_4_)] / N

where N represents the total number of seeds planted, and N_0_–N_4_ represents the average number of seeds/seedlings in groups 0–4, respectively [15].

### 2.3. Effect of Repeated Inoculation on Disease Suppression

The effect of re-inoculation of *S. rolfsii, R. solani* and BNR was examined. Two weeks after planting radish in repeatedly infected soil with *R. solani*, *S. rolfsii*, and BNR, or sterile barley grains, the disease was assessed. The experiment was carried out exactly as described previously. The experiment was conducted three times, with three replicates in each treatment and assessed similarly.

### 2.4. PCR-DGGE Analysis of the Inoculated Soil 

#### 2.4.1. DNA Extraction from Soil 

Soil samples were collected and stored at −20 °C until use. Soil was disrupted using crushing equipment (Fast Prep FP series, 5.5 m/sec., 30 s). DNA was extracted from soil using Fast DNA Spin Kit (Q-BioGene Inc., Tokyo, Japan) according to the recommended protocol. 

#### 2.4.2. Analysis of Microflora

For microflora analysis, a primer set targeting the V6-8 variable region of eubacterial 16S rDNA gene was designed (F984GC: 5′-GC clamp-aa cgc gaa gaa cct tac -3′R1378:5′-cgg tgt gta caa ggc ccg gga acg-3’) and used [29]. A KOD-Plus (Toyobo Life Science, Tokyo, Japan) kit was used in PCR. The reaction solution contained: 32 μL sterile distilled water, 5.0 μL 10 × PCR buffer, 5.0 μL 2 mM dNTP, 2.0 μL 25 mM MgSO_4_, 1.0 μL 10 μM F984GC, 1.0 μL 10 μM R13781, 2.0 μL 10 mg/ml BSA, 1.0 μL 1.0 U/μL KOD-Plus and 1.0 μL DNA template, and the total volume was adjusted to 50 μL. The PCR was amplified under the conditions of 34 cycles (94 °C for 15 s, 55 °C for 30 s and 68 °C for 30 s). The amplification product was purified using a GeneElute^TM^ PCR Clean-UP Kit (SIGMA-ALDRICH Inc., Tokyo, Japan). After purification, DNA concentration was measured using Nano Vue and stored at −20 °C. The DGGE was performed using a Dcode^TM^ Universal Mutation Detection System (Bio-Rad Laboratories) according to the Dcode manual: 6% acrylamide gel: bis acrylamide (37.5:1) was set to 50–70%. PCR-amplified material was loaded onto the DGGE gel and subsequently electrophoresed at 58 °C at a constant voltage of 50 V for 18 h run in 1 × TAE. The gel was stained for 30 min using SYBR green I. The DGGE bands were photographed using Typhoon 9400. The experiment was repeated three times. 

#### 2.4.3. Analysis of Filamentous Fungus 

For filamentous fungi analysis, the following primer set targeting the 18S rRNA gene was designed and used according to May et al. [30] (NS1: 5′-gta gtc ata tgc ttg tct c-3′, GCFung: 5′-GC clamp-at tcc ccg tta ccc gtt g -3′). A KOD-Plus (Toyobo Life Science, Tokyo, Japan) kit was used in PCR. The reaction solution contained: 31 μL sterile distilled water, 5.0 μL 10 × PCR buffer, 5.0 μL 2 mM dNTP, 2.0 μL 25 mM MgSO_4_, 1.5 μL 10 μM NSI, 1.5 μL 10 μM GCFung, 2.0 μL 10 mg/mL BSA, 1.0 μL 1.0 U/μL KOD-Plus and 1.0 μL Template DNA, and the total volume was adjusted to 50 μL. The PCR was amplified under the conditions of 30 cycles (94 °C for 15 s, 50 °C for 30 s and 68 °C for 30 s). The amplification product was purified using a GeneElute^TM^ PCR Clean-UP Kit (SIGMA-ALDRICH Inc., Tokyo, Japan). DNA concentration was measured using NanoVue (GE Healthcare) and then stored at −20 °C. The DGGE was performed using A Dcode^TM^ Universal Mutation Detection System according to the Dcode manual and the 7% acrylamide gel: bis acrylamide solution (37.5:1) was set to 20–45%. The PCR-amplified material was loaded on the DGGE gel, which was subsequently electrophoresed at 60 °C using a constant voltage of 50 V for 20 h run in 1 × TAE. Gel was stained for 30 min using SYBR green I (Invitrogen). The DGGE bands were photographed using Typhoon 9400 (GE Healthcare). The experiment was repeated three times. 

Bands in the gel were cut and transferred to a 1.5 mL tube. Bands were triturated with a vortex and 750 μL of TE was added. DNA was purified by shaking for 1 h at 37 °C, then centrifuged for 7 min at 13,000 rpm, and the supernatant was ethanol precipitated. Primers were used without GCclamp, DGGE analysis, and PCR amplification was conducted as previously described. The nucleotide sequence of the obtained DNA fragment was carried out according to the recommended protocol by using reaction sequence Big DyeR Terminatar v3.1 cycle sequencing kit (ABI, Inc. Tokyo, Japan) and Genetic Analyzer PRISM3100. The BLAST was determined based on the nucleotide sequence [31]. The sequence was subjected to an homology search using the International Nucleotide Sequence Database (GeneBank/DDBJ). The experiment was repeated three times.

### 2.5. Quantification and Frequency of Trichoderma Species by Soil Dilution Plate Technique

#### 2.5.1. Quantification of *Trichoderma* Species in the Inoculated Soil

Populations of *Trichoderma* were calculated in the soil before and after each time of inoculation. Sterile water (45 mL) was used to suspend soil samples (5 g) with shaking (200 rpm for 30 min), which were then serially diluted (10^–3^ ~ 10^–5^). Three replicates were used for each sample by plating 100 μL of the suspension onto 10 mL agar on Petri dishes followed by incubation for 4 days at 25 °C. The number of *Trichoderma* colonies was then counted. *Trichoderma* selective medium contained TSM: 1000 mL distilled water, 0.9 g K_2_HPO_4_, 0.2 g MgSO_4_ 7H_2_O, 1.0 g NH_4_NO_3_, 0.15 g KCL, 0.15 g D-Glucose, 0.15 g Rose bengal, 50 mg Chloramphenicol, 20 mg Streptomycin, 0.2 g PCNB, 0.3 g P-DASS and 20 g Agar as recommended by Elad et al. [32]. The quantity of *Trichoderma* spp. in the soil was measured according to the following equation [15]:

Cfu / (g dry soil) = (number of colonies × dilution factor) × (soil fresh weight / soil dry weight)

#### 2.5.2. Frequency of *Trichoderma* in the Inoculated Soil

After counting *Trichoderma* colonies on TSM, 20 colonies were randomly selected, implanted on PDA medium and cultured for 30 days at 25 °C. Representative isolates were cultured on potato dextrose broth (PDB) for 10 days at 25 °C. Thereafter, cells were collected using an aspirator and stored at −20 °C. After rapid freezing of 100 mg of the culture with liquid nitrogen, samples were ground in a mortar and pestle. DNA was extracted with the PEX extraction method [33]. Specific primers for the ITS region were designed according to White et al. [34] (ITS1:5′-tccgtaggtgaacctgcgc-3′, ITS4: 5′-tcctccgcttattgatatgc-3′). A PCR was performed using the following reaction solution; 12.9 μL of sterile distilled water, 2.0 μL of 10 × PCR buffer, 2.0 μL of 2 mM dNTP, l.0 μL of 10 μM ITS1, 1.0 μL of 10 μM ITS4, 0.1 μL of 5 U/μL Ex Taq and 1.0 μL of DNA template, and the total volume was adjusted to 20 μL. DNA was amplified under the conditions of 30 cycles (94 °C for 15 s, 50 °C for 30 s and 68 °C for 30 s). The amplification product was purified using a GeneElute^TM^ PCR Clean-UP Kit. The obtained DNA fragment was determined by using nucleotide sequence according to the recommended protocol, with a Big Dye^R^ Terminatar v3.1 cycle sequencing kit and Genetic Analyzer PRISM3100. The BLAST was determined based on the nucleotide sequence [30]. The sequence was subjected to an homology search in the International Nucleotide Sequence Database (GeneBank/DDBJ). The homology was high. Furthermore, 20 strains were isolated from each experimental plot and the ratio of each species was calculated.

### 2.6. Statistical Analysis

XLSTAT PRO statistical analysis software was used to separate the means by Duncan’s multiple range test (DMRT, *p* ≤ 0.05). Using Fisher’s LSD test, we were able to differentiate the treatment means for at least three separate experiments. EKUSERU-TOUKEI 2010 (SSRI Co., Ltd., Tokyo, Japan) was used to perform a Steel–Dwass test. *p* ≤ 0.05 was used as the significance level for all analyses.

## 3. Results

### 3.1. Disease Severity 

Disease severity values were significantly reduced in soil that was repeatedly inoculated with *R. solani* and *S. rolfsii*. Disease severity of sterile barley grain was 0.24, 0.21 and 0.35 in the first, second and third inoculations, respectively (Table 1). Meanwhile, *R. solani* exhibited low disease severity values from the first (3.89) through the third inoculation (1.33). Similarly, disease severity values in soil repeatedly inoculated with *S. rolfsii* were 3.97, 2.34 and 1.38 in the first, second and third inoculations, respectively (Table 1). Disease decline was not observed using BNR (Table 1). Based on the above results, disease decline was confirmed due to repeated inoculations with *S. rolfsii* and *R solani*.

### 3.2. Suppression of Radish Root Rot Disease

Soil that was repeatedly inoculated three times with sterile barley grains served as control. Disease severity was 4.00 in the control treatment for *S. rolfsii* and *R. solani* (Table 2). Repeated inoculations of soil with *R. solani* significantly reduced the disease severity of *R. solani* and *S. rolfsii* to 1.40 and 1.70, respectively. On the other hand, disease severity of *R. solani* was 4.00 in the soil repeatedly inoculated with *S. rolfsii*, which indicated that the disease severity of *R. solani* was not suppressed at all, while the disease severity of *S. rofsii* was significantly decreased to 1.50 in soil repeatedly inoculated with *S. rolfsii* (Table 2). Soil inoculated three times with BNR showed the same tendency as the soil with repeated *R. solani* inoculations, recording 1.20 and 0.90 for *S. rolfsii* and *R. solani*, respectively (Table 2).

### 3.3. PCR-DGGE of Microbial Population

**Figure 1:**
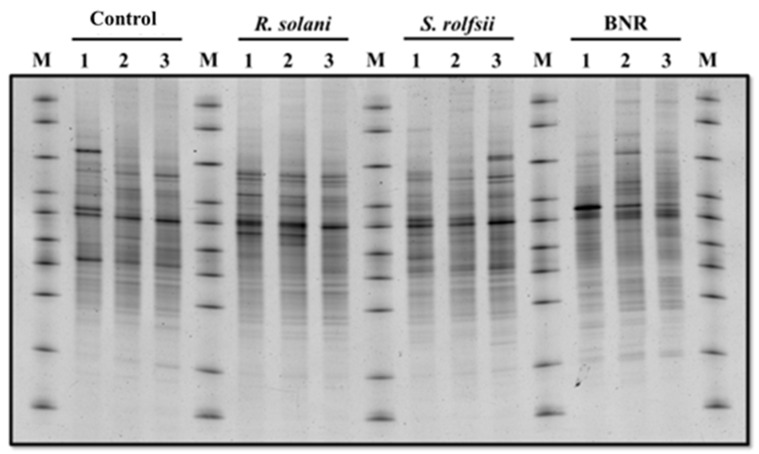
PCR-DGGE band patterns of microbial flora associated with the repeated inoculation of non-pathogenic binucleate Rhizoctonia, *Rhizoctonia solani* and *Sclerotium rolfsii*. M = marker; 1 = first time repeated inoculation; 2 = second time repeated inoculation; 3 = third time repeated inoculation.

**Figure 2:**
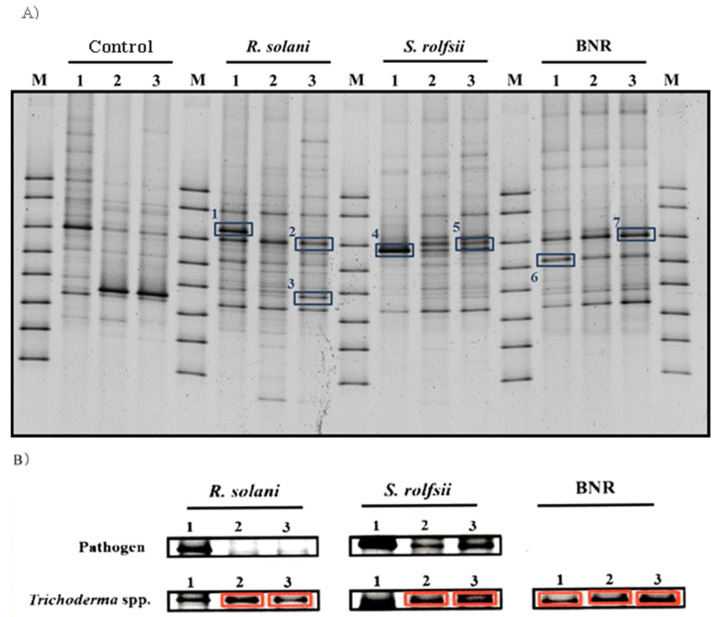
PCR-DGGE band patterns of filamentous fungi phase due to repeated inoculation of non-pathogenic binucleate *Rhizoctonia*, *Rhizoctonia solani* and *Sclerotium rolfsii* (**A**). PCR-DGGE band patterns of filamentous fungi phase due to repeated inoculation of BNR, *R. solani* and *S. rolfsii.* Changes in the band pattern of *Trichoderma* spp. and pathogens associated with repeated inoculation of BNR, *R. solani* and *S. rolfsii* (**B**). (**A**): M = marker; *R. solani* (Band 1); *Trichoderma* sp. (Band 2); *Cunninghamella* sp. (band 3); *S. rolfsii* (Band 4), *Trichoderma* sp. (Band 5); *Penicillium* sp. (Band 6) and *Trichoderma* sp. (Band 7). (**B**): 1 = first time repeated inoculation; 2 = second time repeated inoculation; 3 = third time repeated inoculation.

DGGE bands of microbial community structure were observed in repeatedly inoculated soil. Figure 1 shows the band patterns of 1–3 inoculations of soil with BNR, *R. solani* and *S. rolfsii*, compared with band patterns of 1–3 inoculations of soil with sterile barley grains (control). No noticeable changes were observed in the band patterns between all treatments and the control (Figure 1). On the other hand, DGGE band patterns were observed in soil repeatedly inoculated with BNR, *S. rolfsii* and *R. solani* but not in the control treatment (Figure 2A). Seven major bands were found in the homology search after decryption of the nucleotide sequence (Table 3). In the repeatedly inoculated soil with *R. solani,* bands of *R. solani* (Band 1) and *Trichoderma* sp. (Band 2) were observed. After the third inoculation time, *Cunninghamella* sp. (Band 3) was seen, a fungus that is well isolated from the soil as saprotrophs. Similarly, in repeated inoculation soil with *S. rolfsii*, bands of *S. rolfsii* (Band 4) and *Trichoderma* sp. (Band 5) were observed. In repeatedly inoculated soil with BNR, *Penicillium* sp. (Band 6) and *Trichoderma* sp. (Band 7) were observed. However, the band of BNR was not found (Figure 2A).

*Trichoderma* sp. was reported in all treatments. To confirm the changes in the band patterns of *S. rolfsii* and *R. solani*, all the bands present in the same position were cut, and an homology search was conducted after decryption of the nucleotide sequence. Figure 2B clearly illustrated the changes in the band patterns of *Trichoderma* sp. and pathogens in repeatedly inoculated soil. In the pathogen bands, *R. solani* was seen in the first inoculation but not in the second and third inoculations. In the case of *S. rolfsii*, the band was darker in the first inoculation and thin bands were observed in the second and third inoculations. Bands of *Trichoderma* sp. were observed in the second and third inoculations with *S. rolfsii* and *R. solani* and all inoculation times with BNR. *Fusarium* sp. was found in the first inoculation with *R. solani* using sequence analysis. Further bands were cut out of the first inoculation with *S. rolfsii* and we attempted to decipher the base sequence, but it could not be decoded (Figure 2B).

### 3.4. Quantification and Frequency of Trichoderma Species in Repeatedly Inoculated Soils 

#### 3.4.1. Quantification of *Trichoderma* Species in Repeatedly Inoculated Soils 

The quantities of *Trichoderma* spp. in the first, second and third inoculations of soil with sterilized barley grains (control) were 1.4 × 10^4^ cfu/g, 7.2 × 10^4^ cfu/g and 6.2 × 10^4^ cfu/g dry soil, respectively (Table 4). On the other hand, the quantity of *Trichoderma* species was 3.7 × 10^4^ cfu/g, 14.8 × 10^4^ cfu/g and 10.1 × 10^4^ cfu/g dry soil in the first, second and third inoculations of soil with *R. solani*, respectively (Table 4). A similar trend was also observed in the soil repeatedly inoculated with *S. rolfsii*; the quantity of *Trichoderma* spp. was significantly increased in the second and third inoculations. Although the quantity of *Trichoderma* spp. was slightly reduced in the third inoculation, it was still significant compared with the control group (Table 4). In contrast, a significant increase in the quantity of *Trichoderma* spp. was observed in the first inoculation with BNR, and it reached 21.1 × 10^4^ cfu/g dry soil. After the second inoculation, the quantity of *Trichoderma* spp. was increased to 32.9 × 10^4^ cfu/g dry soil, and finally, it was further decreased to 23.6 × 10^4^ cfu/g dry soil in the third inoculation. Thus, the repeated inoculation of BNR revealed variation patterns of *Trichoderma* spp., which were different from those of *S. rolfsii* and *R. solani* (Table 4).

#### 3.4.2. Frequency of *Trichoderma* spp. in Repeatedly Inoculated Soils

**Figure 3:**
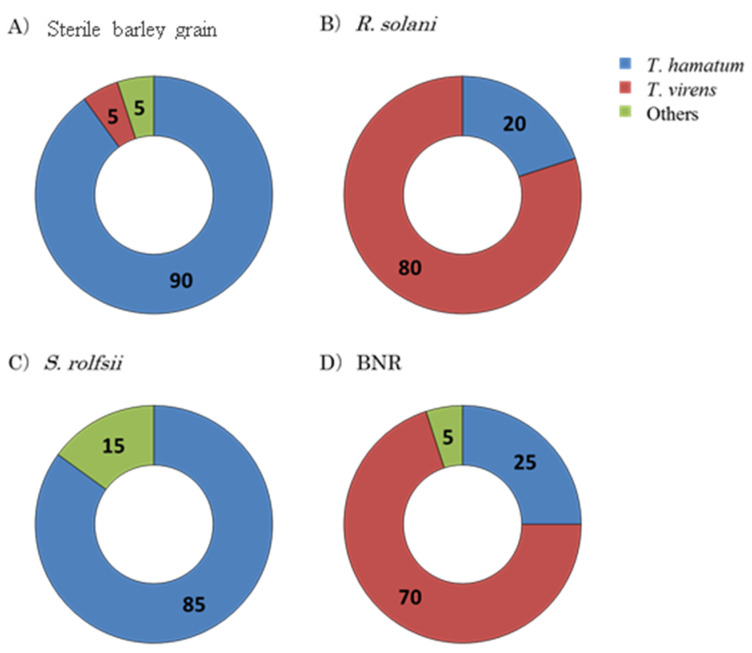
Soil dilution plate technique of non-pathogenic *Rhizoctonia*, *Rhizoctonia solani* and *Sclerotium rolfsii.* The percentages of frequency of the genus *Trichoderma* in soil repeatedly inoculated with *R. solani,* binucleate *Rhizoctonia* (BNR) and *S. rolfsii*. (**A**) Soil was repeatedly inoculated three times with sterile barley grain; (**B**) Soil was repeatedly inoculated three times with *R. solani*; (**C**) Soil was repeatedly inoculated three times with *S. rolfsii*; (**D**) Soil was repeatedly inoculated three times with BNR.

*Trichoderma* spp. isolated from repeatedly inoculated soil were examined by analyzing the nucleotide sequence of the ITS region for identifying the species composition of the Trichoderma (Figure 3).

As a result, the frequencies of *Trichoderma* spp. in soil repeatedly inoculated with sterile barley grains were 90% *T. hamatum*, 5% *T. virens* and 5% of the other remaining species. On the other hand, the frequency of *Trichoderma* species in soil repeatedly inoculated with *R. solani* was 80% *T. virens* and 20% *T. hamatum*. Lastly, in *S. rolfsii*-inoculated soil, *T. virens* was not detected at all, and the ratio of *T. hamatum* was the highest (85%). Repeated inoculations of soil with BNR exhibited accumulation patterns similar to *R. solani*-inoculated soil, 70% *T. virens* and 25% *T. hamatum*.

## 4. Discussion

*Rhizoctonia solani* is a very common pathogen in most soils with a great diversity of host plants [35]. It is a worldwide pathogen causing severe damage to many economically important crops [35]. It also causes root rot disease on radish seedlings, which is a very destructive disease. Economic losses are evident due to the importance of quality seedling production. *Sclerotium rolfsii* is a soilborne fungal pathogen that causes disease in a wide range of plants [27]. Serious crop losses occur every year due to *S. rolfsii* infection. The ability to produce persistent sclerotia, and the broad host range of *S. rolfsii* contribute to the major crop losses for farmers worldwide.

Disease decline due to the introduction of living inocula was demonstrated by previous reports [4,8,13,36]. In this study, individual treatments had different patterns of disease severity across the three inoculations. Soils repeatedly inoculated with *S. rolfsii* and *R. solani* caused consistent disease decline compared to the control. Meanwhile, soil repeatedly inoculated with BNR did not initiate disease decline after any of the three inoculations. The results indicated that the repeated inoculation of soil with BNR and *R. solani* significantly suppressed both *S. rolfsii* and *R. solani* , while the repeated inoculation of soil with *S. rolfsii* significantly suppressed only *S. rolfsii*.

The dynamics of pathogen inoculations by the PCR-DGGE method were investigated. The band of *R. solani* was observed in the first inoculation but not in the second and third inoculations. On the other hand, the band of *S. rolfsii* was dark in the first inoculation and thin in the second and third inoculations. Fungal mycelia, spores and bacteria (microbial biomass) are considered the main components of soil organics [37]. Disease severity values after three successive inoculations with *S. rolfsii*, *R. solani* and BNR reflect the contribution of general biota on the formation of suppressive soils. The results of the dilution plate technique showed that *Trichoderma* spp. was accumulated in the repeatedly inoculated soil with BNR, *S. rolfsii* and *R. solani*. Additionally, the PCR-DGGE results exhibited that the bands of *Trichoderma* sp. were observed in the repeatedly inoculated soil with *R. solani, S. rolfsii* and BNR, which is consistent with the results of the dilution plate method. However, it is difficult to use quantitative analysis techniques such as the dilution plate technique for *S. rolfsii* and *R. solani* because they do not form spores. *Trichoderma* spp. were significantly increased in soil inoculated repeatedly with BNR from the first to third inoculations. Thus, repeated inoculation of soil with BNR revealed variation in the quantity and distribution of *Trichoderma* which is different from that of *R. solani* and *S. rolfsii*. It is believed that the repeated inoculations of BNR are more effective in promoting the accumulation of indigenous *Trichoderma* than *S. rolfsii* and *R. solani*. The frequencies of *Trichoderma* spp. in soils repeatedly inoculated with *S. rolfsii*, *R. solani* and BNR were estimated. *T. virens* and *T. hamatum* were the most accumulated species from the genus of *Trichoderma* in the repeatedly inoculated soil with BNR and *R. solani*. *T. hamatum* was also accumulated in the repeatedly inoculated soil with *S. rolfsii,* while *T. virens* was rarely isolated. Selective accumulation of *Trichoderma* spp. occurred due to the difference in the type of inoculum in repeatedly inoculated soil. Thus, *Trichoderma* species are important for the disease suppression. *Trichoderma* spp. were reported as antagonists to different soil-borne pathogens that occur naturally [38,39] and have already been commercialized as biocontrol agents against different pathogens [39]. Pathogen mycelia and/or sclerotia may be invaded by species of *Trichoderma*, reducing the formation of new propagules [10]. *Trichoderma* spp. are also known to generate antifungal enzymes, including chitinases that have direct and indirect effects on the cell membranes of the target fungi [40]. For example, the production of lytic enzymes by *T. hamatum* was reported against *S. rolfsii* and *R. solani* [40].

## 5. Conclusions

The findings showed that inoculating soil with BNR and *R. solani* multiple times substantially reduced both *S. rolfsii* and *R. solani* , whereas inoculating soil with *S. rolfsii* multiple times greatly suppressed only *S. rolfsii*. *Trichoderma* spp. were found to play an important role in disease suppression in repeatedly inoculated soil. The species of *Trichoderma* probably grew over the inocula of the pathogens in the successive treatments and were continued with the new colonies that were added at each consecutive inoculation. A further study is necessary to elucidate the selective accumulation mechanisms that control the difference in the quantity of *Trichoderma* spp. due to repeated inoculation of the pathogen. These findings could help establish a stable biocontrol method of root rot pathogens.

## Figures and Tables

**Table 1:** Changes in radish root rot caused by repeated inoculation of non-pathogenic binucleate *Rhizoctonia*, *Rhizoctonia solani* and *Sclerotium rolfsii*.

Treatment	Disease Severity ^1^		
	Number of Inoculation Times		
	1	2	3
Control	0.24 ± 0.08a ^2^	0.21 ± 0.08a	0.35 ± 0.10a
*R. solani*	3.89 ± 0.07a	2.71 ± 0.20b	1.33 ± 0.14c
*S. rolfsii*	3.97 ± 0.03a	2.34 ± 0.31b	1.38 ± 0.16c
BNR	0.27 ± 0.08a	0.19 ± 0.10a	0.35 ± 0.15a

^1^ Numbers are means ± standard error of the severity of radish root rot disease. ^2^ Different alphabets indicate significant differences between treatments (Steel–Dwass test, *p* < 0.01).

**Table 2:** Suppression of radish root rot disease due to soil repeatedly inoculated three times with the non-pathogenic binucleate *Rhizoctonia* (BNR), *Rhizoctonia solani* and *Sclerotium rolfsii*.

Treatment	Disease Severity ^1^			
	Repeatedly Inoculated Soil			
	Control	*R. solani*	*S. rolfsii*	BNR
*R. solani*	4.00 ± 0.00a ^2^	1.40 ± 0.15b	4.00 ± 0.00a	0.90 ± 0.25b
*S. rolfsii*	4.00 ± 0.00a	1.70 ± 0.06b	1.50 ± 0.06b	1.20 ± 0.20b

^1^ Numbers are means ± standard error of the severity of radish root rot disease. ^2^ Different alphabets indicate significant differences between treatments (Steel–Dwass test, *p* < 0.01).

**Table 3:** Homology search results of DGGE fragments of filamentous fungi phase due to repeated inoculation of non-pathogenic binucleate *Rhizoctonia*, *Rhizoctonia solani* and *Sclerotium rolfsii*.

Band	Base Sequence Length (bp)	Homology Search Results (DDBJ BLAST Search)		
		Species Name	Percentage of Homology (%)	Access No.
1	267	*Rhizoctonia solani*	100	D85641
2	309	*Trichoderma* sp.	100	AF406811
3	219	*Cunninghamella* sp.	93	JF824699
4	288	*Sclerotium rolfsii*	100	JF819726
5	211	*Trichoderma* sp.	83	AJ783947
6	292	*Penicillium* sp.	97	GU733355
7	275	*Trichoderma* sp.	100	DQ310767

**Table 4:** Changes in the quantity of *Trichoderma* spp. due to repeated inoculation of binucleate *Rhizoctonia*, *Rhizoctonia solani* and *Sclerotium rolfsii* using the soil dilution plate technique.

Treatment	*Trichoderma* spp. Quantity (×10^4^ cfu/g Dry Soil) ^1^		
	Number of Inoculation Times		
	1	2	3
Control	1.4 ± 0.2c	7.2 ± 0.2a	6.2 ± 0.2b
*R. solani*	3.7 ± 0.4c	14.8 ± 0.6a	10.1 ± 0.8b
*S. rolfsii*	2.3 ± 0.1c	14.0 ± 0.3a	10.3 ± 0.1b
BNR	21.1 ± 0.5b	32.9 ± 0.6a	23.6 ± 0.3b

^1^ Numbers are means ± standard error of the quantity of *Trichoderma* spp.

## Data Availability

Available upon request from the corresponding author.
